# Heterogeneity Analyzed by CT‐Based Habitat Analysis for Clinical Management of Cancers: A Narrative Review

**DOI:** 10.1002/kjm2.70257

**Published:** 2026-06-25

**Authors:** Yang Zhang, Zhi‐Yan Jin, Min‐Ying Lydia Su

**Affiliations:** ^1^ Department of Radiological Sciences University of California Irvine California USA; ^2^ Zhejiang Cancer Hospital, Hangzhou Institute of Medicine (HIM), Chinese Academy of Sciences Hangzhou China; ^3^ Department of Medical Imaging and Radiological Sciences Kaohsiung Medical University Kaohsiung Taiwan

**Keywords:** cancer management, CT, habitat, machine learning, radiomics

## Abstract

Tumors are highly heterogeneous, and whole‐lesion radiomics analysis is a popular method for extracting texture features that reflect this heterogeneity, which can be used to build models for cancer diagnosis, staging, therapy response evaluation, and prognosis prediction. However, there is no spatial information for understanding which components provide the most critical information. Recently, habitat analysis has gained widespread attention, and it is used to divide a tumor into distinct sub‐regions, called habitats, that share common characteristics. CT reveals heterogeneity in attenuation, texture, entropy, and enhancement patterns, which can be used to generate habitats. In the past 2 years, many studies exploring the role of CT habitats have been reported. The divided habitats can be used to build models for the classification of histological or molecular subtypes, the evaluation and prediction of treatment response, and prognostication of progression‐free survival or overall survival. This review aims to summarize studies on the application of CT‐habitat analysis for cancer management. In addition to clinical applications, various methods for habitat segmentation will be reviewed. Lastly, how spatial information revealed by the habitat can guide tissue biopsy for tissue‐level confirmation studies will be described, which is critical for understanding the underlying biology and paving the way for future clinical implementation.

## 1 Introduction

In the last decade, MRI‐based habitat analysis has been extensively performed. Multi‐parametric MRI can provide information on water content, edema, vascularity, cellular density, etc., and many studies have investigated its application in cancer management. In contrast to conventional whole‐tumor analysis, habitat analysis aims to divide a lesion into multiple subregions with relatively homogeneous imaging characteristics, thereby preserving spatial information that may better reflect tumor biology and microenvironmental diversity, which are known to be associated with pathological types, aggressiveness, treatment sensitivity, and long‐term prognosis. CT is another imaging modality commonly applied for various cancers. The habitats can be generated using attenuation values, texture features, entropy measures, enhancement patterns, dual‐energy parameters, or multimodal PET/CT data combined with PET metabolic information. In the past 2 years, many CT studies across different tumor types have been published. The habitat‐derived descriptors have been applied to a wide range of clinical tasks, including diagnosis, risk staging, molecular/genetic subtype characterization, treatment response prediction, and prognosis stratification [1, 2, 3, 4]. After habitats are generated, they serve as the basis, usually for building binary classification models.

A notable trend in the recent literature is that habitat analysis is rarely used on its own, such as by using components or relative percentages from segmented habitats to make classifications. Instead, the segmented habitats are often used to extract radiomics features. Compared with conventional radiomics analysis performed on the whole lesion, the number of generated features was multiplied by the number of segmented habitats, providing more flexibility for feature selection and often resulting in better classification performance. Furthermore, the developed habitat radiomics models can be combined with clinical variables and biological markers to further improve predictive performance and support clinical translation [3, 5, 6, 7]. In addition to the intratumoral analysis, the peritumoral region is often generated to obtain information from the tumor boundary and the surrounding microenvironment, which are known to contain clinically important features related to invasion, immune response, and treatment resistance [8, 9, 10, 11].

**FIGURE 1:**
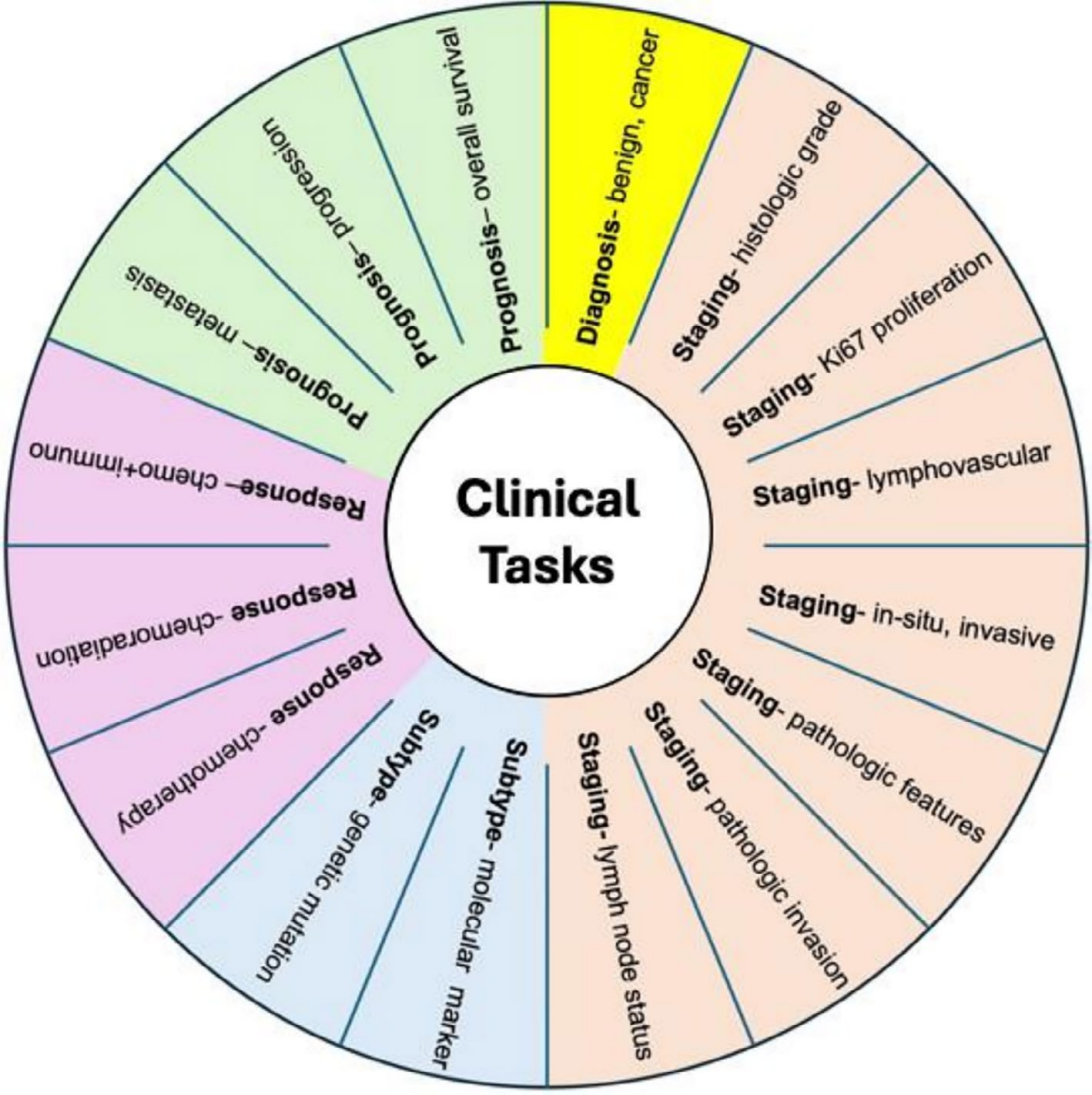
An overview of the clinical applications.

The reported clinical applications can be categorized into several areas, including (1) differential diagnosis of benign and malignant diseases, (2) staging based on histological grades/features, involved lymph nodes, and other aggressive biomarkers such as Ki‐67 and lymphovascular invasion, (3) subtype characterization based on molecular and genetic biomarkers for choosing targeted therapy, (4) prediction of neoadjuvant treatment response, (5) stratification of treatment outcomes or prognosis based on development of metastasis, specific disease‐free survival, progression‐free survival, or overall survival. An overview of the clinical applications is shown in Figure 1.

In Section 2, the reported clinical applications of CT‐based habitat analysis across various tumors are summarized. It will be organized by body regions, starting with lung cancer, since it is the most comprehensive and covers all applications, followed by anatomical regions from head/neck to pelvis. The summary of the reviewed studies, the clinical applications, and a brief description of the specific task performed in each study are listed in Table 1. In Section 3, several major habitat segmentation and analysis methods are described, summarized in Table 2. In Section 4, the clinical implementation and future research directions are provided.

**TABLE 1:** Summary of clinical applications in diagnosis, staging, subtype characterization, treatment response evaluation, and outcome stratification.

Ref [number], year	Clinical application	Specific tasks
2.1. *Lung cancer*		
[1] Aminu et al. 2022	Diagnosis and prognosis	Cancer patients infected with COVID‐19
[5] Wang et al. 2025	Prognosis—PFS	Progressive‐free survival of ALK‐positive lung cancer
[6] Wu et al. 2025	Prognosis—PFS	PFS and immune‐related adverse reaction
[9] Dong et al. 2025	Staging	Differentiate in situ/minimally invasive vs. invasive cancer
[10] Shang et al. 2024	Staging	Prediction of lung adenocarcinoma invasiveness
[12] Chen et al. 2021	Diagnosis	Discriminate lung cancer vs. benign inflammation
[13] Zuo et al. 2024	Diagnosis	Benign and malignant masses in COPD background
[14] Peng et al. 2024	Staging	Predict spread through air spaces in stage T1 cancer
[15] Huang et al. 2025	Staging	Predict micropapillary/solid components in stage I
[16] Xu et al. 2025	Staging	Predict lymphovascular invasion in T1‐stage lung cancer
[17] GYe et al. 2024	Therapy response	Predict pCR to neoadjuvant immunochemotherapy
[18] Ye et al. 2025	Therapy response	Optimize neoadjuvant chemoimmunotherapy decision
[19] Wu et al. 2024	Subtype characterization	Predict EGFR mutation status in stage I lung cancer
[20] Ji et al. 2025	Subtype characterization	Classify PD‐L1 expression in locally advanced lung cancer
[21] Sujit et al. 2024	Prognosis—recurrence	Predict recurrence, integrative radiogenomics‐blood insights
[22] Li et al. 2025	Staging	Predict invasiveness and grade
2.2. *Oral, laryngeal, and nasopharyngeal carcinoma*		
[23] Dong et al. 2025	Staging	Predict Ki‐67‐positivity in laryngeal squamous cancer
[24] Yin et al. 2025	Therapy response	Response to chemoradiotherapy in nasopharyngeal cancer
[25] Liu et al. 2025	Staging	Predict cervical lymph node metastasis in oral cancer
2.3. *Thyroid cancer*		
[26] Feng et al. 2025	Staging–risk stratification	Identify low‐risk papillary thyroid carcinoma
[27] Shen et al. 2026	Diagnosis	Differentiate papillary thyroid carcinoma from nodular goiter
2.4. *Thymic epithelial tumors*		
[28] Wang et al. 2025	Staging–risk stratification	High‐risk [B2/B3/thymic carcinoma] vs. low‐risk [A/AB/B1]
[29] Yang et al. 2025	Staging–risk stratification	Predict thymic epithelial tumors risk categorization
[30] Liang et al. 2025	Staging–risk stratification	Differentiate between high and low‐risk thymomas
[31] Liu et al. 2024	Staging–risk stratification	Assess risk categorization of thymomas
2.5. *Esophageal cancer*		
[11] Ling et al. 2026	Therapy response	Predict pCR after neoadjuvant chemoimmunotherapy
[32] Peng et al. 2025	Therapy response	Prediction of post‐neoadjuvant lymph node metastasis
[33] Kong et al. 2024	Therapy response	Predict response to neoadjuvant chemoimmunotherapy
[34] Zhang et al. 2025	Response and prognosis	Forecast pCR and progression‐free survival (PFS)
[35] Gao et al. 2026	Response and prognosis	Predict treatment response and long‐term survival
2.6. *Hepatocellular carcinoma*		
[3] Shen et al. 2025	Therapy response	After transarterial chemoembolization + immunotherapy
[4] Zhang et al. 2025	Prognosis	Prediction of early postoperative recurrence
[36] Chen et al. 2025	Prognosis	Predict survival and immune status in HCC
[37] Zhao et al. 2026	Prognosis—recurrence	Early recurrence prediction and risk stratification in HCC
[38] Wu et al. 2025	Therapy response	Predict response to hepatic artery infusion chemotherapy
[39] Tang et al. 2024	Subtype characterization	Predict Tim‐3 immune checkpoint expression
2.7. *Cholangiocarcinoma*		
[40] Chen et al. 2025	Staging–risk stratification	Predict lymph node metastasis in cholangiocarcinoma
2.8. *Pancreatic ductal adenocarcinoma*		
[2] Song et al. 2025	Staging	Predict pancreatic adenocarcinoma pathological grading
2.9. *Renal cancer*		
[7] Li et al. 2025	Staging	Predict Ki‐67 proliferation in clear cell renal cell carcinoma
[8] Chen et al. 2025	Staging	Predict WHO/ISUP grading in clear cell renal cell carcinoma
[41] Shan et al. 2025	Prognosis	Prediction of PFS in clear cell renal cell carcinoma
[42] Yang et al. 2025	Prognosis	Predicting metastasis risk in clear cell renal cell carcinoma
2.10. *Bladder cancer*		
[43] Du et al. 2025	Staging	Predicting muscle invasion in bladder cancer
[44] Du et al. 2025	Staging	Predict muscle invasion using a dual‐energy CT
[45] Du et al. 2025	Staging	Preoperative prediction of muscle invasion in bladder cancer
[46] Du et al. 2025	Staging	Assessment of tumor stromal heterogeneity
[47] Li et al. 2025	Prognosis	Post‐operative recurrence in non‐muscle invasive bladder Ca
2.11. *Ovarian cancer and cervical cancer*		
[48] Liu et al. 2025	Therapy response	Neoadjuvant chemotherapy in high‐grade serous ovarian Ca
[49] Wang et al. 2022	Staging and prognosis	Ki‐67 status and PFS in high‐grade serous ovarian cancer
[50] Mu et al. 2020	Prognosis	Predict chemoradiotherapy outcome in cervical cancer
2.12. *Colorectal cancer and colorectal metastases*		
[51] Su et al. 2025	Staging	Assessment of lymphovascular invasion in colorectal cancer
[52] Zhao et al. 2024	Subtype characterization	Classification of KRAS/NRAS/BRAF mutation status
[53] Huang et al. 2024	Therapy response	Radiofrequency ablation in colorectal cancer lung metastases
[54] Zhou et al. 2025	Prognosis	PFS, overall survival, in colorectal cancer liver metastases
2.13. *Stroke*		
[55] Jiang et al. 2025	Therapy response	Predict stroke source, response to machine thrombectomy

**TABLE 2:** Summary of habitat analysis methods and their clinical applications across different diseases.

Habitat generation and analysis methods	References
3.1 Manual tumor ROI segmentation	Hepatocellular carcinoma [4] Laryngeal cancer [23]
3.2 Peritumoral area expansion	Hepatocellular carcinoma [4] Renal cancer [7, 8] Lung cancer [9, 10, 14, 19] Esophageal cancer [11] Thyroid cancer [27] Thymomas [31] Colorectal cancer [53]
3.3 Intensity‐based K‐means clustering	Pancreatic ductal adenocarcinoma [2] Hepatocellular carcinoma [3, 38, 39] Lung cancer [5, 6, 9, 14, 15, 16, 17, 19] Renal cancer [7, 8, 42] Oral cancer [25] Thyroid cancer [26, 27] Thymic epithelial tumors [28, 29] Thymoma [30, 31] Esophageal cancer [32] Cholangiocarcinoma [40] Bladder cancer [45] Ovarian cancer [48] Colorectal cancer [53] Stroke [55]
3.4 Texture features‐based clustering	Lung cancer [13] Esophageal cancer [33, 34] Renal cancer [41] Bladder cancer [43, 44, 46, 47] Ovarian cancer [49, 56] Colorectal cancer [54]
3.5 Otsu thresholding	Ovarian cancer [48] Hepatocellular carcinoma [36] Cervical cancer [50]
3.6 PET/CT features‐based clustering	Lung cancer [12, 20, 21] Esophageal cancer [35] Ovarian cancer [48, 49] Cervical cancer [50] Colorectal cancer [51, 52]
3.7 Nested habitat analysis	Hepatocellular carcinoma [37]
3.8 Super‐pixel based clustering	COVID‐19 [1] Lung cancer [18, 21, 22] Nasopharyngeal carcinoma [23, 24] Thyroid cancer [26]
3.9 Intratumoral heterogeneity (ITH) score	Renal cancer [8, 41] Lung cancer [17] Colorectal cancer [51]

## 2 Clinical Applications of Habitat Analysis

### 2.1 Lung Cancer

Lung cancer is highly prevalent with CT as the primary imaging modality, and it is currently one of the most active areas of application for CT‐based habitat analysis. PET imaging is also extensively applied to obtain metabolic information. The reported clinical tasks span diagnostic discrimination, invasiveness assessment, molecular prediction, treatment response evaluation, recurrence risk estimation, and survival stratification, which cover all clinical tasks illustrated in Figure 1. For diagnosis, habitat imaging‐based PET/CT radiomics was shown to help discriminate non‐small cell lung cancer (NSCLC) from benign inflammatory diseases [12]. There are also preliminary applications in the diagnosis of masses arising in the background of chronic lung disease, a diagnostically challenging setting where inflammatory and neoplastic processes overlap [13]. For predicting histologic invasiveness and aggressive pathologic patterns, Dong et al. used CT‐habitat radiomics to differentiate in situ/minimally invasive adenocarcinoma from invasive adenocarcinoma manifesting as ground‐glass nodules [9]. Peng et al. showed that CT‐habitat radiomics improved the prediction of spread through air spaces (STAS) in stage T1 invasive lung adenocarcinoma [14], and Shang et al. demonstrated that a model using tumoral and peritumoral habitat radiomics could predict invasiveness on preoperative chest CT [10]. Huang et al. compared habitat, conventional radiomics, and fusion models for predicting micropapillary/solid components in stage I lung adenocarcinoma [15]. Xu et al. also integrated habitat radiomics and deep learning features to predict lymphovascular invasion in T1‐stage lung adenocarcinoma [16]. As stage‐I lung cancer is usually treated with surgery without adjuvant therapy, identifying patients who have high‐risk factors is important to offer them appropriate therapies to achieve a complete cure.

In the neoadjuvant setting, Ye et al. applied CT‐based quantification of intratumoral heterogeneity to predict pathologic complete response (pCR) to neoadjuvant chemoimmunotherapy in NSCLC [17]. A later multicenter study further extended this concept by integrating longitudinal, multidimensional CT, including habitat imaging, to optimize neoadjuvant chemoimmunotherapy decisions [18]. Determining EGFR status in lung cancer is critical for selecting optimal treatments, and at the molecular level, Wu et al. combined habitat radiomics with deep learning to predict EGFR mutation status in stage I NSCLC [19]. Furthermore, metabolic habitat analysis has been applied to predict PD‐L1 expression in locally advanced NSCLC, with biologically meaningful associations between specific high‐glycolytic habitats and immune‐related gene expression [20]. Such prediction can help select good candidate patients for receiving immunotherapy.

Several studies have focused on recurrence and prognosis. Wang et al. developed a habitat‐based machine learning model for progression‐free survival in ALK‐positive lung cancer [5], while Wu et al. used habitat radiomics to predict both progression‐free survival and immune‐related adverse reactions in NSCLC treated with immunotherapy [6]. PET/CT habitat imaging has been used to identify risk subtypes associated with recurrence in NSCLC and to complement circulating tumor DNA analyses [21]. Explainable PET‐based habitat modeling has also been developed for recurrence prediction in invasive lung adenocarcinoma.

### 2.2 Oral, Laryngeal, and Nasopharyngeal Carcinoma

For head and neck tumors, CT‐habitat analysis has mainly been used to evaluate aggressive biological behavior and treatment response. In laryngeal squamous cell carcinoma, Dong et al. showed that habitat radiomics could improve a nomogram for predicting Ki‐67 positivity [23], suggesting that subregional heterogeneity on CT may reflect proliferative activity. In nasopharyngeal carcinoma, Yin et al. applied tumor habitat‐derived radiomics features on pretreatment CT to predict response to concurrent chemoradiotherapy [24]. For oral cancer, contrast‐enhanced CT feature‐based clustering has been reported as a means of tumor characterization and risk‐related assessment [25].

### 2.3 Thyroid Cancer

Habitat analysis has also shown value in thyroid cancer, particularly in papillary thyroid carcinoma (PTC), where preoperative risk stratification may help identify low‐risk patients suitable for active surveillance rather than undergoing immediate surgery. Feng et al. combined preoperative CT and ultrasound, aiming to identify low‐risk PTC [26]. Multiple habitats were generated from both imaging modalities, and the integrated model incorporating CT‐derived habitat features, ultrasound habitat descriptors, clinical parameters, and immunologic markers achieved excellent performance across validation cohorts [26]. Shen et al. applied CT‐based habitat radiomics, using both intratumoral and peritumoral habitat signatures, for differentiating papillary thyroid carcinoma from benign nodular goiter [27]. Taken together, these studies suggest that CT habitat analysis in thyroid disease may be clinically useful for both diagnostic discrimination and risk‐adapted treatment selection.

### 2.4 Thymic Epithelial Tumors

Thymic epithelial tumors (TETs) are a broad category of rare tumors arising from the thymus, encompassing both slower‐growing, less aggressive thymomas and fast‐growing, highly aggressive thymic carcinomas. While thymomas are the most common type and rarely spread, thymic carcinomas are more aggressive and have a high metastatic potential and significantly lower survival rate. Therefore, accurate preoperative risk stratification is important to guide surgical and oncologic management. Wang et al. demonstrated the diagnostic value of tumor habitat radiomics for risk stratification in thymic epithelial tumors on contrast‐enhanced CT [28]. Yang et al. further combined intratumoral and spatial habitat information with deep learning and radiomics for preoperative prediction of thymic epithelial tumor risk categorization [29]. Liang et al. proposed a CT‐based K‐means habitat segmentation and risk prediction model for thymoma [30], while Liu et al. focused on tumor habitat and peritumoral region evolution‐based imaging features to assess thymoma risk categorization [31].

### 2.5 Esophageal Cancer

**FIGURE 2:**
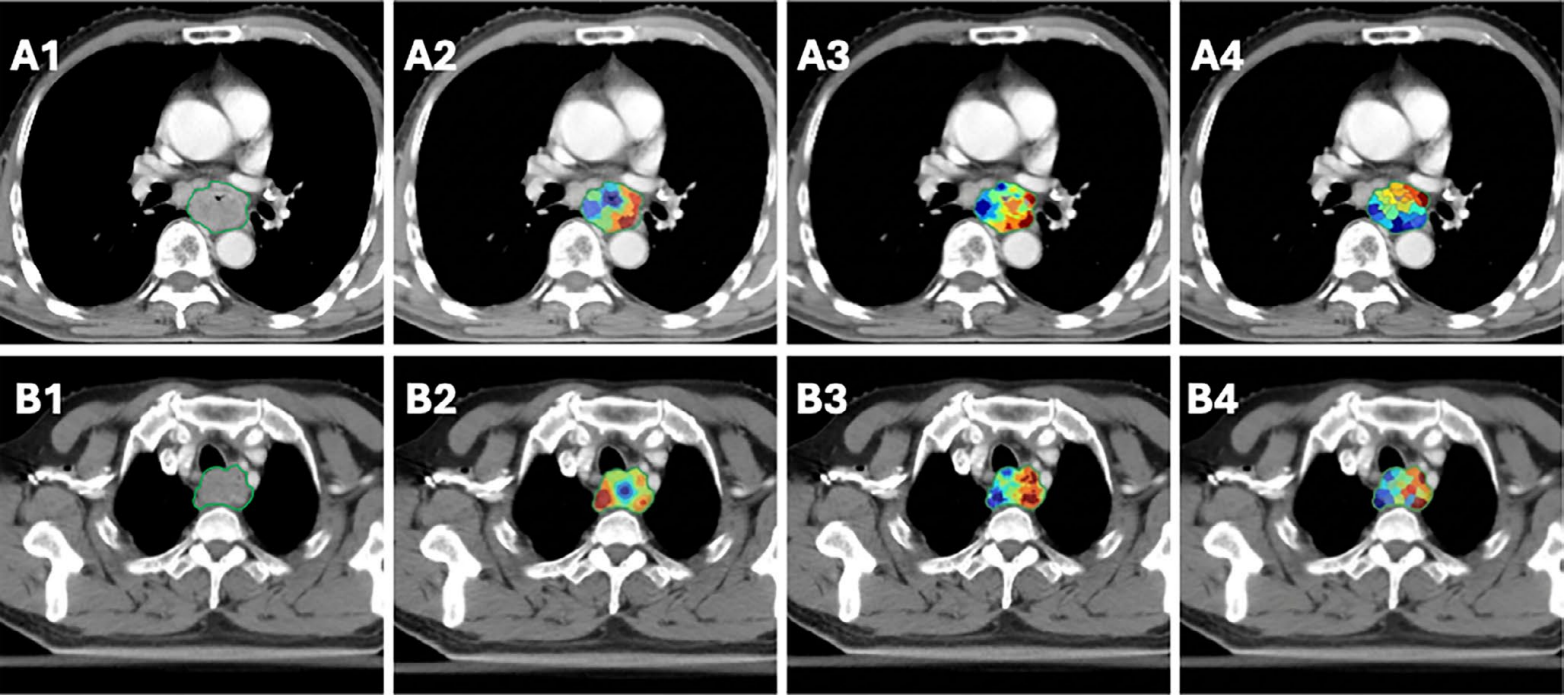
(A) A 66‐year‐old female patient with esophageal squamous cell carcinoma. The patient received IMRT at a total dose of 50 Gy and developed recurrence 15 months later. (A1) Arterial‐phase CT with lesion delineation. (A2) Nested habitat analysis results. (A3) Intensity‐based k‐means clustering results. (A4) Super pixel clustering results. (B) A 75‐year‐old male patient with esophageal squamous cell carcinoma. The patient received IMRT at a total dose of 50 Gy and developed recurrence 9 months later. (B1) Arterial‐phase CT with lesion delineation. (B2) Nested habitat analysis results. (B3) Intensity‐based k‐means clustering results. (B4) Super pixel clustering results.

In esophageal squamous cell carcinoma (ESCC), the critical clinical need is accurate preoperative evaluation of treatment response and residual disease after neoadjuvant therapy. Examples of two patients with esophageal cancer analyzed using three different habitat analysis methods are shown in Figure 2. Peng et al. developed a CT‐habitat radiomics model combined with multi‐instance learning for early prediction of post‐neoadjuvant lymph node metastasis in ESCC [32]. This study is important because it moves beyond primary tumor analysis and addresses nodal residual disease, which has direct implications for surgical planning and prognosis [32]. Kong et al. used CT‐habitat radiomics to predict treatment response to neoadjuvant chemoimmunotherapy [33], while Zhang et al. reported that CT‐habitat could predict both pathologic complete response and survival in ESCC treated by neoadjuvant chemoradiotherapy and esophagectomy [34]. More recently, Ling et al. showed that intratumoral and peritumoral heterogeneity derived from CT can predict pathological response after neoadjuvant chemoimmunotherapy [11]. PET/CT habitat‐radiomics and adaptive deep learning have also been applied to predict treatment response and long‐term survival in ESCC patients undergoing chemoimmunotherapy [35].

### 2.6 Hepatocellular Carcinoma

**FIGURE 3:**
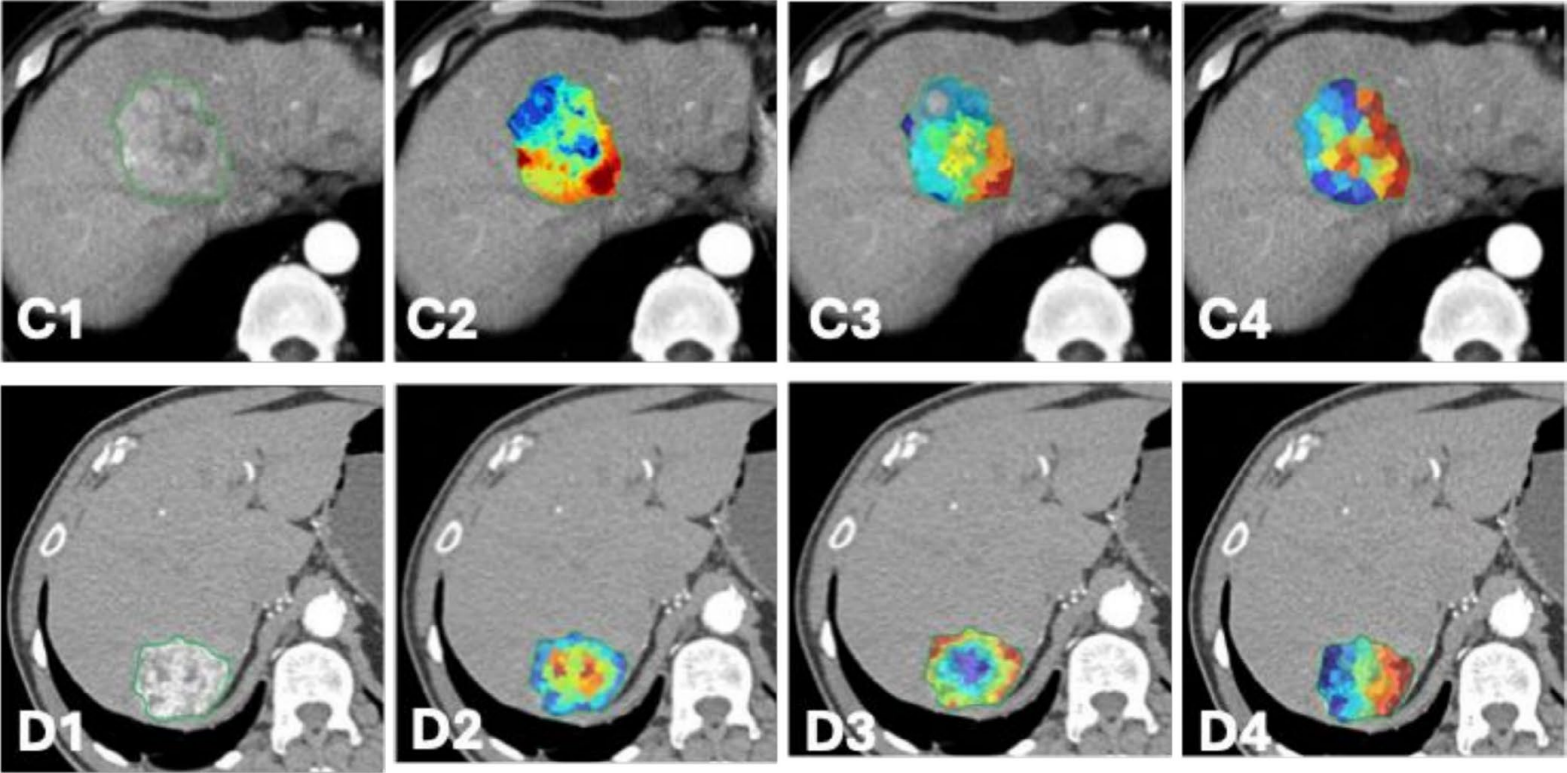
(C) A 58‐year‐old male patient with moderately differentiated HCC. The patient received transarterial chemoembolization and developed recurrence 21 months later. (C1) Arterial‐phase CT with lesion delineation. (C2) Nested habitat analysis results. (C3) Intensity‐based k‐means clustering results. (C4) Super pixel clustering results. (D) A 71‐year‐old male patient with moderately differentiated HCC. The patient received IMRT at a total dose of 54 Gy. (D1) Arterial‐phase CT with lesion delineation. (D2) Nested habitat analysis results. (D3) Intensity‐based k‐means clustering results. (D4) Super pixel clustering results.

Hepatocellular carcinoma (HCC) is one of the tumor types in which CT‐based habitat analysis has been most actively explored. Multi‐phase dynamic contrast‐enhanced CT is often performed at different times, including the arterial phase (35–40 s) for hypervascular tumors, the portal venous phase (70–80 s) providing peak enhancement for parenchyma, and the delayed phase (3–10 min) revealing washout. Examples of two patients with hepatocellular carcinoma analyzed using three different habitat analysis methods are shown in Figure 3. Because liver tumors often exhibit marked spatial heterogeneity in vascularity, necrosis, viable tumor components, and the peri‐tumoral microenvironment, habitat analysis is particularly well suited to this disease. Shen et al. developed a CT‐habitat model to predict tumor response and overall survival in unresectable HCC treated with transarterial chemoembolization combined with molecularly targeted agents and immune checkpoint inhibitors, and showed that habitat outperformed conventional whole‐tumor radiomics for both response prediction and survival stratification [3]. Zhang et al. applied habitat analysis based on different phases of contrast‐enhanced CT to predict early postoperative recurrence of HCC [4]. Because recurrence is strongly influenced by intratumoral aggressiveness and microvascular invasion‐related biology, subregional analysis may provide more biologically relevant information than global descriptors [4]. Chen et al. further showed that CT habitat radiomics could predict survival and immune status in HCC across multiple cohorts [36]. In addition, more advanced CT‐based habitat approaches have been proposed, including nested habitat analysis and contrast‐enhanced feature‐based clustering, further expanding the methodological landscape in HCC [37, 38, 39]. Overall, the HCC literature indicates that habitat analysis can contribute to prognostic stratification, recurrence prediction, response assessment, and possibly characterization of the immune microenvironment.

### 2.7 Cholangiocarcinoma

Mass‐forming intrahepatic cholangiocarcinoma presents substantial heterogeneity and carries a high risk of lymph node metastasis. Chen et al. used CT‐radiomics features derived from tumor habitat subregions to predict lymph node metastasis [40]. Habitat‐based subregional analysis may help better characterize the invasive front or biologically aggressive components that are not adequately represented by whole‐lesion averages [40].

### 2.8 Pancreatic Ductal Adenocarcinoma

Pancreatic ductal adenocarcinoma (PDAC) is one of the most aggressive solid malignancies, and reliable preoperative assessment of tumor grade is important for treatment planning and prognostic evaluation. Song et al. developed a CT‐habitat radiomics model combined with topological analysis to predict pathological grading [2]. The portal venous phase CT images were segmented into three habitat regions by K‐means clustering, and radiomics and topological features were extracted from each subregion. The results showed that the final Habitat model outperformed both the clinical model and the whole‐tumor radiomics model and suggested that spatial subregions with different attenuation characteristics may correspond to distinct stromal or cellular components, thereby improving preoperative risk stratification.

### 2.9 Renal Cancer

Clear cell renal cell carcinoma (ccRCC) risk stratification is another important clinical setting where both intratumoral and peritumoral habitat information may help. Li et al. integrated multi‐scale radiomics and deep learning for Ki‐67 prediction in ccRCC and found that habitat radiomics constituted an important predictive component [7]. Chen et al. used intratumoral and peritumoral habitat imaging for preoperative prediction of WHO/ISUP grade in ccRCC [8], and Shan et al. reported a clinic‐radiomics model based on intratumoral habitat imaging to predict progression‐free survival [41]. Additionally, contrast‐enhanced CT habitat approaches have also been applied to predict metastasis risk in ccRCC [42].

### 2.10 Bladder Cancer

Bladder cancer is another tumor type in which CT‐habitat analysis has been actively applied, especially to predict whether it is muscle‐invasive or not, which is a critical factor for choosing the optimal therapy. Du et al. performed a multicenter contrast‐enhanced CT habitat radiomics study to predict muscle invasion [43]. The dual‐energy CT‐habitat models also showed promising performance for muscle invasion prediction and interpretability [44, 45]. Later work further integrated habitat radiomics with deep learning to assess tumor stromal heterogeneity and predict not only recurrence‐free survival but also CD8+ T‐cell infiltration and immunotherapy response [46]. Li et al. coupled habitat radiomic analysis with the concept of tumor ecosystem diversification to assess postoperative recurrence in non‐muscle invasive bladder cancer [47].

### 2.11 Ovarian Cancer and Cervical Cancer

In gynecologic malignancies, PET/CT‐based habitat analysis has shown promise for both treatment response prediction and biologic characterization. In high‐grade serous ovarian cancer, Liu et al. used intratumoral habitat heterogeneity on 18F‐FDG PET/CT to predict early response to neoadjuvant chemotherapy [48]. An earlier study also demonstrated that PET/CT habitat radiomics could predict Ki‐67 status and progression‐free survival in ovarian cancer [49]. These applications are clinically meaningful because ovarian cancer is highly heterogeneous and has a poor prognosis, and a noninvasive stratification of proliferative activity and treatment sensitivity could help guide management. In cervical cancer, PET/CT features‐based habitat clustering has also been applied to outcome‐related prediction [50].

### 2.12 Colorectal Cancer and Colorectal Metastases

Although MRI is the main imaging modality for colorectal malignancies, there are a few CT studies. Su et al. quantified intratumoral heterogeneity using habitat analysis for preoperative assessment of lymphovascular invasion in colorectal cancer [51]. Zhao et al. applied habitat‐derived radiomic analysis using 18F‐FDG PET/CT to predict KRAS/NRAS/BRAF mutations [52]. In a metastatic setting, habitat‐based radiomics has also been used to evaluate the immediate response of colorectal cancer lung metastases treated with radiofrequency ablation [53], and to predict progression survival and overall survival in patients with liver metastases [54]. These findings suggest that habitat analysis may be useful not only in primary colorectal cancer but also in metastatic disease, where lesion heterogeneity may influence local control and survival.

### 2.13 Stroke and Other Emerging Non‐Neoplastic Applications

**FIGURE 4:**
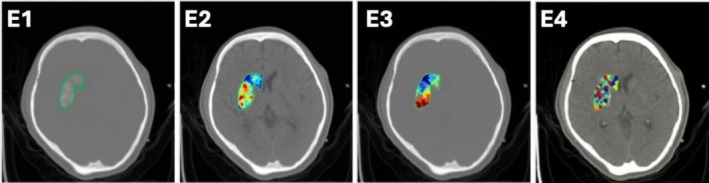
(E) A 50‐year‐old male patient with cerebral hemorrhage. (E1) Arterial‐phase CT with lesion delineation. (E2) Nested habitat analysis results. (E3) Intensity‐based k‐means clustering results. (E4) Super pixel clustering results.

Beyond oncology, some studies have applied dual‐energy CT habitat approaches to non‐neoplastic diseases such as stroke [55]. Such work suggests that habitat analysis is a general imaging paradigm for spatial heterogeneity assessment and may continue to expand into other disease domains. An example of a patient with intracerebral hemorrhage analyzed using three different habitat analysis methods is shown in Figure 4. Jiang et al. [55] used dual‐energy CT (DECT) to assess thrombotic heterogeneity and constructed models based on whole‐thrombus and subregional radiomics features for predicting stroke source and clinical outcome after mechanical thrombectomy. Their results showed that DECT outperformed conventional CT, and thrombus subregion analysis provided the highest predictive performance. In addition, the identified DECT‐based subregions were correlated with histopathologic thrombus composition, suggesting that habitat analysis may offer a visual and biologically meaningful approach for characterizing thrombus constituents.

## 3 Habitat Segmentation and Analysis Methods

Habitat segmentation on CT aims to partition a tumor and, in some studies, its surrounding tissue into multiple spatially distinct subregions that are relatively homogeneous with respect to attenuation, texture, enhancement pattern, entropy, metabolic activity, or other derived imaging properties. Compared with conventional whole‐tumor radiomics, this strategy attempts to preserve regional heterogeneity rather than averaging it away. The methodological workflow includes several common steps: delineation of the tumor region of interest (ROI) or volume of interest (VOI), optional generation of peritumoral extensions, determination of voxel‐ or subregion‐level descriptors for analysis, clustering or threshold‐based subdivision into habitats, extraction of habitat‐specific features, and finally model construction using machine learning or deep learning methods, mostly for binary classification as shown in Figure 1. The major segmentation methods are summarized below, and listed in Table 2.

### 3.1 Manual Tumor ROI Segmentation

The most fundamental method is manual delineation of the tumor ROI before further habitat partitioning. The lesion is first contoured on CT images by radiologists or trained readers, and the delineated tumor is then used as the analysis space for habitat generation or direct subregional feature extraction. This approach remains the most common starting point because it is intuitive and clinically interpretable.

The major advantage of manual ROI segmentation is that it provides anatomically constrained lesion definition, which is particularly important when tumors are irregular or adjacent to structures that may confound automated segmentation. In addition, it allows subsequent habitat generation to focus specifically on biologically meaningful intratumoral heterogeneity. However, this method is also limited by observer dependence, variable contouring standards across centers, and the labor‐intensive nature of slice‐by‐slice annotation. These issues may affect the reproducibility of habitats, especially in multicenter studies where acquisition protocols and image quality vary widely.

### 3.2 Peritumoral Area Expansion

The extension to the peritumoral region is for analysis of the tumor boundary and surrounding tissues. In this strategy, after the primary tumor is delineated, the contour is expanded outward by a fixed distance, such as 1, 2, 4, 5, 8, or 10 mm, to include surrounding tissue that may harbor infiltration, stromal reaction, immune response, vascular remodeling, or subtle tumor‐microenvironment interaction. They may provide complementary information on invasion and the stromal microenvironment as they relate to therapy response. For example, in lung cancer, peri‐tumoral habitats have been associated with invasiveness, spread through air spaces, mutation prediction, and response to neoadjuvant treatment. In renal cancer and thymoma, intratumoral and peritumoral habitats have similarly been shown to improve grading or risk classification. The main methodological challenge is that the optimal expansion distance is not standardized and may vary by organ, lesion size, local anatomy, and the clinical question. Moreover, larger expansions increase the risk of including irrelevant normal tissue.

### 3.3 Intensity‐Based K‐Means Clustering

One of the most straightforward habitat generation methods is intensity‐based clustering, usually implemented with K‐means. Here, voxel intensities within the tumor are used directly to divide the lesion into subregions with different attenuation profiles. Intensity‐based clustering is attractive because it is simple, computationally efficient, and closely linked to the CT signal. Attenuation differences may reflect necrosis, fibrosis, hemorrhage, cellular density, or vascular heterogeneity, depending on the tumor type and enhancement phase. In practice, K‐means clustering is commonly used to generate two to five subregions, although the optimal number of clusters varies by study. The method is easy to implement and remains interpretable, but it is also relatively coarse because it relies on intensity alone and may not fully capture more subtle textural or biologic differences. As a result, intensity‐based clustering often serves as either an initial partitioning strategy or a benchmark against which more advanced methods are compared.

### 3.4 Texture‐Feature‐Based Clustering

A more advanced habitat‐generation strategy uses texture and entropy features, rather than raw voxel intensity alone, to define subregions. In this framework, local image descriptors are first calculated to characterize spatial variation, structural complexity, and regional disorder within the lesion, and clustering is then performed in the derived feature space to generate habitats. Compared with intensity‐based partitioning, this approach can better capture subtle intratumoral heterogeneity that may not be apparent on visual inspection or conventional attenuation analysis. The major strength of this approach is that it provides a richer representation of tumor organization. Texture features can describe local gray‐level variation, spatial arrangement, coarseness, and directional heterogeneity, whereas entropy maps emphasize the degree of randomness or disorder within different tumor regions. These properties may better reflect biologic complexity, including variable cellularity, stromal composition, necrosis, and infiltrative growth patterns. The texture‐ and entropy‐feature‐based clustering lies between simple signal partitioning and fully model‐driven habitat generation. As it relies on processed maps, careful standardization is essential when applying this method in multicenter studies.

### 3.5 Otsu Thresholding

Otsu thresholding is a straightforward segmentation method, in which the image histogram is automatically partitioned into discrete intensity groups by maximizing between‐class variance. Compared with iterative clustering, Otsu thresholding is simpler and less computationally demanding. It is especially useful when the image distribution naturally separates into distinct attenuation or metabolic states. The limitation is that threshold‐based methods assume separable intensity classes and may be less flexible in tumors with continuous or overlapping signal distributions. However, this method is transparent, easily interpretable, and relatively robust.

### 3.6 PET/CT‐Feature‐Based Clustering

PET/CT‐based habitat analysis integrates anatomic information from CT with metabolic information from PET. In these studies, habitat generation is typically based on FDG uptake, CT attenuation, or combined PET/CT descriptors, and the resulting subregions are then used for prognosis, treatment response prediction, or molecular inference. The key strength of PET/CT habitats is their biologic richness. For example, high‐glycolytic or metabolically active subregions may correspond to aggressive clones, hypoxic niches, or immune‐evasive habitats.

### 3.7 Nested Habitat Analysis

Nested habitat analysis is one of the most conceptually advanced methods in the current literature. Rather than performing a single partition of the tumor, this strategy iteratively identifies higher‐risk subregions within already defined habitats, thereby creating a hierarchical or multilevel map of aggressiveness. The nested framework is especially appealing because it aligns with the biological intuition that aggressive tumors may contain hotspots within hotspots rather than a flat set of equal subregions. In practice, the method often begins with a whole‐tumor radiomics model that generates a probability map or risk map, after which clustering is repeatedly applied to isolate increasingly aggressive habitats. This type of recursive focusing may improve early recurrence prediction and risk stratification, as shown in HCC [37]. Methodologically, nested habitat analysis moves beyond descriptive partitioning toward progressive biological refinement, but it also introduces more parameters and potential overfitting risk, making external validation particularly important.

### 3.8 Super‐Pixel Based Clustering

Super‐pixel based clustering is a spatially constrained strategy for habitat generation. Instead of clustering individual pixels or voxels directly, the tumor ROI is first re‐sampled into multiple small and locally homogeneous regions, usually referred to as super‐pixels. Each super‐pixel contains adjacent pixels with similar intensity, texture, or structural appearance. Quantitative descriptors can then be extracted from each super‐pixel, and these local units are further grouped into larger habitat subregions. This strategy has been used in multimodal habitat imaging for papillary thyroid carcinoma [26], pretreatment CT‐based habitat analysis for nasopharyngeal carcinoma [24], and PET/CT‐based NSCLC recurrence prediction, where features were extracted from super‐pixels [21]. In lung adenocarcinoma, ternary‐classification habitat modeling for subsolid nodules also supports the broader value of local subregion‐based habitat analysis for characterizing subtle intratumoral heterogeneity [22].

The main advantage of this method is that it preserves local spatial organization while reducing pixel‐level noise. Compared with voxel‐wise clustering, super‐pixels provide more stable analytical units and can be integrated with radiomics, deep learning, or multi‐instance learning frameworks. However, the resulting habitats are sensitive to the number, size, and compactness of super‐pixels, and these parameters remain insufficiently standardized. Future studies should further evaluate parameter stability, three‐dimensional implementation, multicenter reproducibility, and biological correlation with pathology or spatial molecular data.

### 3.9 Intratumoral Heterogeneity (ITH) Score

The intratumoral heterogeneity score is designed to summarize the spatial diversity of tumor imaging phenotypes into a compact quantitative biomarker. In this framework, the tumor is first divided into local analytical units such as pixels, voxels, patches, super‐pixels, or subregions. Local radiomics or intensity‐based descriptors are then calculated, and these units are grouped into different imaging phenotypes. The final ITH score reflects the composition, diversity, distribution, or spatial organization of these phenotypes within the tumor. Recent studies have applied ITH score‐based or ITH‐related models for WHO/ISUP grade prediction in clear cell renal cell carcinoma [8], prediction of spread through air spaces in stage T1 invasive lung adenocarcinoma [14], and metastasis risk stratification in clear cell renal cell carcinoma [42].

Compared with conventional habitat radiomics, the ITH score provides a more direct and interpretable measurement of tumor heterogeneity. A higher score may reflect greater phenotypic diversity, uneven habitat distribution, or more irregular spatial organization, which may be associated with clonal diversity, hypoxia, necrosis, angiogenesis, stromal remodeling, immune heterogeneity, and treatment resistance. However, there is currently no universally accepted definition or calculation formula for imaging‐derived ITH. The score may also be affected by tumor size, image resolution, segmentation accuracy, and the number of predefined phenotypes. Therefore, future studies should focus on biological validation, test–retest stability, and reproducibility across centers, scanners, and tumor types.

## 4 Clinical Implementation and Future Research

As reviewed in this article, most CT‐habitat studies have been published in the past 2 years (2024 to 2025), indicating that this is an emerging research field. The current literature indicates that CT‐based habitat analysis has evolved into a versatile framework with broad clinical applications across many tumor types. After the habitats are generated, most studies apply radiomics algorithms to derive features, then use machine learning algorithms to select features and build binary classification models. The main tasks can be grouped into several categories. (1) Differential diagnosis to distinguish malignant from benign lesions. However, even if a case is predicted to be benign, biopsy confirmation is usually required. (2) Staging and assessment of aggressiveness‐related biomarkers, including histological grade, Ki‐67, lymphovascular invasion, muscle invasion, in situ vs. invasive, presence of local lymph node metastasis. The binary model is developed to classify cases as more aggressive (e.g., high histological grade, high Ki‐67, presence of lymphovascular invasion, presence of invasion or invasiveness, presence of local nodal metastasis) versus the counterpart of less aggressive. The information may help to choose a more aggressive treatment strategy for high‐risk patients, for example, whether adjuvant therapy is needed after curative surgery. (3) Characterization of subtypes based on molecular biomarkers and genetic mutation status. The binary model is developed to separate cases with positive vs. negative receptors or genetic mutations, which is critical for selecting appropriate targeted therapy. (4) Prediction of treatment response, for example, for neoadjuvant chemotherapy, chemoradiotherapy, chemoimmunotherapy, or locoregional treatments such as ablations. The binary model is developed to predict pCR versus non‐pCR or good vs. poor response, which may inform adjustments to treatment agents or courses. Such models are particularly important, as immunotherapy has proven effective across many cancers, and immuno‐drugs can be added for patients predicted to have a poor response. (5) Prognostic stratification using development of distant metastasis, recurrence, progression‐free survival, and overall survival as outcomes. Patients need to be followed over time; the developed model is used to estimate the probability of progression, and Kaplan–Meier curves can be generated to show the model's performance.

Although these studies have demonstrated the feasibility of developed models for performing these important clinical tasks, clinical implementation remains challenging. For diagnosis, staging, or subtype characterization, tissue confirmation is essential. The need for such models, built using 3‐dimensional imaging of the whole lesion, is often justified by limited biopsy tissue and sampling bias, and by the fact that imaging‐derived features from the whole lesion may provide complementary information. Radiomics models and, more generally, all artificial intelligence image‐based modeling, are referred to as black boxes. Most studies showed that habitat analysis performs better than whole‐tumor radiomics, especially when combined with peritumoral analysis. Beyond offering more features to choose from, the principal value of habitat analysis lies in its ability to preserve spatial heterogeneity rather than compressing the entire lesion into a single set of global descriptors. Habitat analysis may help identify the regions within a tumor that are most informative. It is feasible to perform confirmatory tissue analysis using the identified spatial hotspot to guide biopsy [56, 57], to understand the association with underlying biology and the mechanisms contributing to diagnosis, aggressiveness staging, treatment sensitivity, and long‐term prognosis. Radiogenomics analysis has been widely applied to obtain complementary radiomics and genomics information [58, 59, 60, 61, 62]. Genomic analysis of tissues identified by radiomics habitat can reveal the molecular underpinnings of these imaging subtypes [21]. As CT‐based habitat methodologies continue to mature, their clinical role will likely expand from retrospective model development to more robust multicenter validation, biologic interpretation, and, eventually, clinical decision support. It is highly anticipated that, as more retrospective cases become available, many more CT habitat‐based modeling studies will be published in the near future; however, focusing on understanding and demonstrating the relationship to underlying biology is critical for future clinical implementation.

## Conflicts of Interest

The authors declare no conflicts of interest.

## Data Availability

The data that support the findings of this study are available on request from the corresponding author. The data are not publicly available due to privacy or ethical restrictions.
